# The Role of Transcranial Magnetic Stimulation for the Treatment of Alzheimer’s Disease: A Narrative Review

**DOI:** 10.3390/life16030397

**Published:** 2026-02-28

**Authors:** Vasileios Papaliagkas, Kallirhoe Kalinderi, Maria Moschou, Marianthi Arnaoutoglou, Effrosyni Koutsouraki, Vasileios K. Kimiskidis

**Affiliations:** 1 Department of Biomedical Sciences, International Hellenic University, 57001 Thessaloniki, Greece; roey111@hotmail.com; 2 Department of Clinical Neurophysiology, AHEPA University Hospital, Aristotle University of Thessaloniki, 54636 Thessaloniki, Greece; mar.mos1981@gmail.com (M.M.); marnaoutoglou@yahoo.com (M.A.); 3 1st Department of Neurology, AHEPA University Hospital, Aristotle University of Thessaloniki, 54636 Thessaloniki, Greece; ekoutsou@auth.gr (E.K.); kimiskid@auth.gr (V.K.K.)

**Keywords:** Alzheimer’s disease, transcranial magnetic stimulation, neuromodulation, non-invasive treatment, cognitive decline

## Abstract

Alzheimer’s disease (AD) is the most common neurodegenerative disease that accounts for 60–80% of all dementia cases and affects millions of people worldwide. At present, standard drug therapies provide only limited symptomatic relief. Therefore, the exploration of novel therapeutic approaches is crucial for improving patient outcomes. Transcranial magnetic stimulation (TMS) has emerged as a promising non-invasive neuromodulation technique that may provide benefit in AD management. This review discusses the pathophysiological mechanisms by which TMS operates, evaluates its clinical efficacy in AD patients, assesses its safety profile, and suggests future directions for research.

## 1. Introduction

Alzheimer’s disease (AD) is the most prevalent neurodegenerative disorder and the most common form of dementia, accounting for approximately 60–70% of all dementia cases [1]. It is clinically characterized by progressive cognitive decline that affects memory, executive function, language, and visuospatial abilities, ultimately leading to loss of functional independence. The neuropathological hallmarks of AD are the accumulation of extracellular amyloid plaques that consist of beta-amyloid and intracellular neurofibrillary tangles composed of hyperphosphorylated tau [2,3,4].

Despite decades of research, therapeutic options for AD remain limited. Current pharmacological treatments, such as acetylcholinesterase inhibitors and NMDA receptor antagonists, offer modest symptomatic relief but do not substantially alter disease progression [5]. The recent FDA and EMA approval of the amyloid-targeting disease-modifying therapies donanemab and lecanemab, has shown, until now, limited and often controversial clinical efficacy, highlighting the urgent need for alternative therapeutic approaches that address the complex pathophysiology of AD [6,7].

A growing amount of evidence suggests that AD might be viewed as a disconnection syndrome marked by disrupted synaptic plasticity, impaired excitation–inhibition balance, and large-scale network disruption, particularly within the default mode network (DMN) and hippocampal–prefrontal circuits [8,9,10]. The DMN is a network of interconnected brain regions (hubs) in the medial prefrontal cortex, posterior cingulate cortex or precuneus, and bilateral inferior parietal cortices, as well as supplementary brain regions such as the medial temporal lobes and temporal poles. This network dysfunction precedes structural brain changes and clinical symptoms and is closely associated with cognitive impairment observed in AD [11].

Transcranial magnetic stimulation (TMS) is a non-invasive brain stimulation technique that is capable of directly modulating cortical excitability and inducing long-lasting plastic changes in targeted neural circuits. By generating transient magnetic fields that induce focal electric currents in the cortical tissue, TMS alters brain activity by impacting neuronal firing, synaptic efficacy, and increasing brain network connectivity [12,13]. In particular, repetitive TMS (rTMS) has been shown to induce long-term potentiation (LTP)- or long-term depression (LTD)-like effects and alter network connectivity, likely through modulation of complex mechanisms involving calcium-dependent processes and glutamate receptors [14].

TMS has received FDA clearance for several neuropsychiatric disorders, such as major depression and migraine, and is widely investigated in neurodegenerative diseases [15]. rTMS offers several advantages in the case of AD treatment, i.e., it directly targets disrupted neural circuits, engages activity-dependent plasticity mechanisms, modulates inflammatory processes, and might influence beta-amyloid and tau protein cascades through network-driven clearance mechanisms and metabolic regulation [16,17,18].

Over the last twenty years, an increasing number of clinical studies have evaluated the use of rTMS as a therapeutic approach in AD, using various stimulation protocols, target regions, and outcome measures. The most common stimulation site for rTMS is the dorsolateral prefrontal cortex (DLPFC); however, recent studies have adopted multisite, DMN-based, and personalized targeting strategies, including stimulation of posterior cortical hubs such as the precuneus [19,20,21,22]. The heterogeneity of protocols, however, limits the generalizability of efficacy findings, and reported outcomes have been inconsistent. This discrepancy highlights the need for careful protocol selection, patient stratification, and standardized outcome measures.

Moreover, preclinical and translational studies have started to elucidate the molecular and cellular mechanisms underlying TMS-induced effects in AD animal models. These involve modulation of synaptic plasticity pathways, regulation of neuroinflammatory responses mediated by microglia and astrocytes, enhancement of neurotrophic signaling, and improvements in neurovascular coupling and metabolic function [23,24,25].

Recent advances in fluid biomarkers, in particular plasma phosphorylated tau217 (p-Tau217), allow reliable identification of amyloid-positive individuals across the Alzheimer’s disease continuum, even at preclinical and prodromal stages. This allows the possibility of rTMS, guided by biological disease staging that targets patients during early phases, when synaptic and network dysfunction are potentially reversible and neuromodulation might be most effective. The aim of this review is therefore not merely to summarize existing clinical studies, but to provide a neurophysiological and biomarker-informed framework for rTMS in AD, by integrating molecular, cellular, network, and clinical evidence, while highlighting both the current limitations and potential directions for clinical application.

## 2. Materials and Methods

This study is a narrative literature review with a structured methodology. A search of the PubMed and Scopus databases was conducted for studies published between 2015 and 2025 using the keywords: “Alzheimer’s disease”, “transcranial magnetic stimulation”, and “rTMS”. Studies were eligible for inclusion only if they included at least 10 participants.

## 3. Principles of Transcranial Magnetic Stimulation

### 3.1. Biophysical Basis of TMS

TMS is based on Faraday’s law of electromagnetic induction. A rapidly changing current passing through a stimulation coil of wire generates a magnetic field that penetrates the scalp and skull and induces an electric field in the underlying neural and non-neural tissues, leading to depolarization or hyperpolarization of neuronal membranes. [26]. The physiological effects of TMS depend on multiple factors, such as stimulation frequency and intensity, coil geometry, number of pulses, and the functional state of the targeted cortex. Although TMS is applied on a specific site, its effects extend to distant interconnected brain regions through anatomical and functional connectivity, resulting in modulation of large-scale networks. Therefore, the beneficial effects of TMS may be the result of the stimulation of the targeted cortical area, the propagation to interconnected regions, or the combined effect of both, as a function of white matter connectivity [27].

While the physical principles of TMS are well established, their relevance to AD physiology lies in the fact that a primary pathophysiological hallmark of the disease is synaptic and network dysfunction rather than neuronal loss alone. AD is characterized by early impairment of LTP, altered excitation–inhibition (E/I) balance, and cortical hyperexcitability; mechanisms that can be modulated by TMS application.

#### 3.1.1. Magnetic Field Generation and Induced Currents

A circular or figure-of-eight coil in TMS produces strong, transient magnetic fields, typically 1–2 Tesla, with a pulse width of 80 to 300 microseconds. The shape and size of the TMS coil play an important role in determining focality and depth of the brain stimulation. The pyramidal neurons in the cortex are more easily excited when the stimulating electric currents flow in parallel to the axons of the pyramidal neurons, compared with the stimulation by currents that are perpendicular to the axons [28]. The induced current is attenuated as a function of distance by bone, air, tissues, and possible structural alterations of the cortex. Layer-5 pyramidal neurons are particularly sensitive due to their exceptionally long apical dendrites that reach all six layers of cortex and are aligned with the induced current [29].

#### 3.1.2. Neuronal Activation Mechanisms

TMS pulses mainly stimulate axonal initial segments and dendritic branches, generating action potentials that propagate in both orthodromic and antidromic directions within cortical networks. This depolarization triggers synaptic release of neurotransmitters and initiates downstream neuroplasticity mechanisms. rTMS modulates cortical excitability through activity-dependent synaptic plasticity; in particular, intrinsic plasticity mechanisms that include: (a) amplification or attenuation of postsynaptic potentials at the dendrites; (b) alterations in the resting membrane potentials; and (c) an increase in the threshold required for the generation of action potentials [30]. rTMS may influence GABA and glutamate levels (the principal inhibitory and excitatory neurotransmitter in the CNS respectively), enhancing GABAergic neuron function and GABA expression. In particular, LF-rTMS (1 Hz), intermittent TBS, and continuous TBS were shown to influence the GABAergic circuitry of the rat cortex [31].

#### 3.1.3. Field Orientation and Cortical Geometry

Cortical folding morphology and orientation influence TMS-induced electric fields. Neurons aligned parallel to the induced electrical field (E-field) are preferentially activated, while those perpendicular may require higher stimulation intensity. Computational modeling of individualized head anatomy and cortical gyrification as assessed with brain MRI has demonstrated that E-field strength and directionality correlate with clinical responsiveness, i.e., when the coil orientation is perpendicular to the direction of the sulcus, a greater E-field strength and penetration depth is produced [32].

#### 3.1.4. TMS Protocols and Their Neurophysiological Effects

TMS protocols are highly variable and can be tailored to specific therapeutic goals in AD, which range from modulation of cortical excitability to network-level reorganization. Protocols are generally classified according to frequency, intensity, pattern, and target area, each of which influences neurophysiological responses and subsequent clinical outcomes.

#### 3.1.5. Frequency-Dependent Effects

- High-frequency TMS (>5 Hz) typically initiates facilitation effects by increasing cortical excitability and inducing long-term potentiation (LTP)-like effects mediated by NMDA receptor activation, calcium influx, and downstream signaling pathways involving BDNF and CREB; pathways that are impaired in AD. In AD patients, high-frequency stimulation of the dorsolateral prefrontal cortex (DLPFC) or precuneus enhances synaptic plasticity, functional connectivity, and cognitive performance, particularly in memory and executive domains [23,24,25,26,27,28,29,30,31,32,33]. rTMS studies in AD patients are almost exclusively performed with high-frequency protocols (>10 Hz). In a recent review [34], HF-rTMS was found to improve global cognitive function in patients with mild to moderate AD as assessed with MMSE score, ADAS-Cog and P300 latency.
- Low-frequency TMS (≤1 Hz) generally induces inhibitory effects on motor cortical excitability and is associated with long-term depression (LTD)-like effects. LF rtMS of 0.5 Hz inhibited the initiation of early action potentials in AD mice [35]. In particular, targeting hyperactive parietal regions, such as the precuneus and the posterior parietal cortex, can restore network balance and reduce excitotoxicity [23,33].

#### 3.1.6. Patterned Stimulation Protocols

Recent developments include theta-burst stimulation (TBS), which delivers pulses in bursts of three, at a frequency of 50 Hz and an inter-burst interval in the theta-frequency range of 200 ms (5 Hz). The two most frequent patterns used are intermittent (iTBS) and continuous (cTBS), which can induce rapid LTP- or LTD-like changes. iTBS has been shown to enhance hippocampal–prefrontal connectivity and memory encoding in mild cognitive impairment and early AD [36], while cTBS results in inhibitory effects. The advantage of TBS is shorter session duration and lower stimulation intensities, making it clinically feasible for repeated interventions [37].

#### 3.1.7. Intensity and Pulse Number

Stimulation intensity is typically expressed as a percentage of resting motor threshold (RMT) and determines the depth and magnitude of cortical engagement. Studies in AD patients have used 80–120% RMT to achieve adequate excitatory effects without inducing adverse events. Higher cumulative pulses per session are associated with stronger and longer-lasting neuroplastic changes, although tolerability should be monitored, particularly in elderly populations.

#### 3.1.8. Target Selection

The target stimulation area of the brain is another important variable that influences neurophysiological and clinical outcomes:

- *Dorsolateral prefrontal cortex (DLPFC):* Enhances working memory, attention, and executive function and increases functional connectivity. It is the most commonly targeted cortical region for AD treatment. 20 Hz rTMS applied to the left DLPFC significantly improves cognitive and psychiatric symptoms in AD patients [38]
- *Precuneus and posterior cingulate cortex (PCC):* Both sites are key nodes of the default mode network (DMN); their stimulation improves episodic memory and network connectivity. In particular, the effects of rTMS in the precuneus might propagate in the hippocampus through synaptic transmission in the precuneus-hippocampal pathway [39].
- *Hippocampal network:* Personalized network-targeted TMS improves hippocampal–precuneus functional connectivity and memory consolidation. In particular, rTMS proved to have significant improvement on ADAS-Cog, particularly in the memory domain, as well as S-IADL and the Clinical Dementia Rating Scale–Sum of Boxes (CDR-SOB) scores [33].

Network-based targeting is increasingly preferred over single-region stimulation because it engages distributed circuits underlying cognition and may maximize clinical benefit.

#### 3.1.9. Clinical Application

Developing TMS protocols for AD treatment requires optimizing frequency, intensity, session duration, and target selection to achieve maximal neuroplasticity while minimizing adverse events. What seems to be more effective is to increase session frequency, target brain networks, and combine TMS with cognitive training or medications to produce lasting improvements in cognition and daily functioning.

## 4. Molecular and Cellular Mechanisms of TMS Relevant to Alzheimer’s Disease

**Figure 1:**
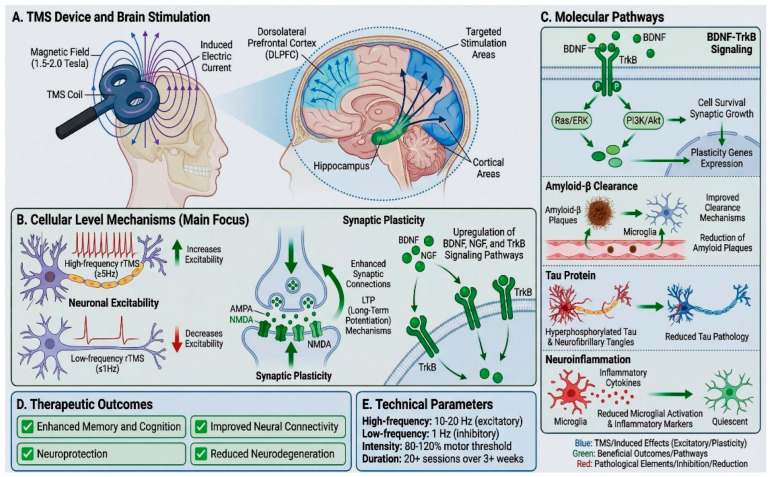
Molecular and cellular mechanisms of TMS and common protocol parameters.

Evidence indicates that TMS influences multiple biological pathways that are involved in the pathophysiology of AD, i.e., enhancement of synaptic plasticity, regulation of inflammation, glial function, network level modulation and beta-amyloid clearance, thus making it a promising technique for modulating the pathophysiology of the disease. These mechanisms are summarized below and are depicted in Figure 1. Preclinical studies in AD animal models suggest that rTMS can influence amyloid and tau pathways; however, there are discrepancies between animal models and human AD pathology, and findings should be interpreted with caution.

### 4.1. Synaptic Plasticity

Synaptic dysfunction can be detected at the early stages of AD pathology even before neuronal degeneration [40]. The induction of brain plasticity requires cell pathways such as the ERK/MAPK signaling pathway and the transcription factor response element-binding protein (CREB) [41]. The pathways are needed to stimulate plasticity-related genes (PRG1-5) and improve cognitive function. Impairment in CREB-mediated gene transcription is associated with beta-amyloid deposition in AD patients. A reduction in CREB, as well as its activated form, pCREB, was observed in the hippocampus and the prefrontal cortex of mice and AD patients, showing the importance of CREB transcription in the pathophysiology of neurodegeneration [42]. A study in a genetic AD mouse model showed that 25 Hz TMS reduced neuronal loss and apoptosis of hippocampal cells due to the activation of PI3K/Akt/GLT-1 pathway, which is associated with decreased excitotoxicity.

Both preclinical and clinical studies demonstrate that rTMS increases brain-derived neurotrophic factor (BDNF) expression in the cortex and hippocampus, as well as peripheral circulation, suggesting systemic effects [31,43]. rTMS also enhances BDNF–TrkB signaling [44] by increasing the affinity of BDNF for TrkB receptor [45]. BDNF-TrkB pathway activates intracellular cascades that promote neuronal survival, growth and plasticity; processes that are severely impaired in AD. TMS might increase BDNF and NGF expression regardless of stimulation frequency, because LF and HF-rTMS both improved their reduced levels in an AD mouse model [46]. However, Choung et al. [47] observed that only HF-rTMS could increase BDNF levels in a mouse model with AD, which was induced by the injection of intracerebroventricular (ICV) Aβ42 oligomer

The presence of a single nucleotide polymorphism BDNF-Val66Met, has also been associated with smaller hippocampal volumes and reduced gray matter in areas of the frontal cortex, as well as with the individual variability of TMS effects [48]. This finding underscores the potential value of prior genetic and molecular testing in optimizing individual TMS protocols.

HF-rTMS induces the expression of the GLUR1 subunit of the AMPA receptors in animal models [49]. As a result, a sufficient number of AMPA receptors are recruited, which causes the opening of NMDA receptors, allowing calcium influx and activating several calcium-sensitive signaling pathways involved in AD pathology [31].

### 4.2. Large Scale Network Modulation

There is evidence that pathophysiological changes observed in AD are associated with both cortical hyperexcitability and functional brain network disruption, particularly involving early changes within the DMN, suggesting that AD might be a disconnection syndrome [50]. TMS can reorganize functional networks such as DMN and hippocampal–prefrontal circuits. In particular, non-invasive stimulation of the precuneus and hippocampal network has been shown to improve connectivity [51,52].

### 4.3. Neuroinflammation and Glial Modulation

Neuroinflammation is involved in the pathophysiology of AD by contributing to synaptic dysfunction, oxidative stress, and, as a result, neuronal loss and network disruption. Moreover, chronic neuroinflammation can cause severe neuronal and blood-brain-barrier disruption. Active glial cells that include microglia and astrocytes, release pro-inflammatory cytokines, complement proteins and reactive oxygen species (ROS), which can cause neuronal damage and lead to AD [53]. rTMS has been shown to modulate the activity of glial cells and inflammatory signaling in models of brain injury and neurodegeneration. Specifically, high-frequency rTMS reduces microglial activation in the dentate gyrus of the hippocampus and suppresses the production of pro-inflammatory mediators (IL-1α, IL-6, TNF-α) and pro-oxidative molecules (ROS and malondialdehyde), while increasing the levels of antioxidants like superoxide dismutase and glutathione in 3xTg AD mice [54]. Astrocytic responses, including regulation of IL-33 expression, further support neuroprotective outcomes. These effects suggest that rTMS could prevent the initiation of the cascade leading to the release of neuroinflammatory products that are involved in AD pathology [55,56].

### 4.4. Effects on Amyloid Metabolism

TMS can indirectly reduce activity-dependent Abeta production by enhancing clearance mechanisms, including microglial phagocytosis and glymphatic flow. High-frequency rTMS stimulation (a) increases expression of BDNF that enhances the activity of a-secretase and reduces Abeta production, and (b) reduces levels of Abeta 1–42 in the hippocampus likely through PI3K/Akt pathway activation via the modulation of BACE1-mediated APP cleavage. rTMS reduces serum levels of Abeta 1–40, Abeta 1–42 and total Abeta [57]. Evidence also shows that rTMS modulates GSK-3β and CDK5 activity kinase/phosphatase pathways that control tau phosphorylation [47,58]. Tao et al. [58] applied 20 Hz rTMS to the DLPFC (100% RMT) for 20 min; after treatment, the soluble ectodomain of p75NTR—which inhibits Abeta accumulation—increased compared to controls.

### 4.5. Neurovascular Regulation and Neurotransmission

Vascular pathology that can exacerbate synaptic failure and cognitive decline plays a significant role in AD pathogenesis [59]. TMS has been shown to increase cerebral blood flow and induce angiogenesis [60]. Enhanced perfusion and vascular reactivity may improve neuronal resilience against the accumulation of toxic metabolites. Moreover, rTMS of the prefrontal cortex can induce the release of dopamine, improving metabolic efficiency within the targeted networks [61]. McNerney et al. [62]. studied the effects of HF-rTMS (10 Hz, applied daily for 2 weeks or twice weekly for 6 weeks) in 3xTgAD mice and showed that rTMS increases acetylcholine levels by modulating AChE activity. In preclinical models, chronic rTMS treatment was found to modulate cortical serotonergic, adrenergic, and dopaminergic circuits in the rat brains [63]. These vascular and metabolic effects complement synaptic and glial modulation, and current evidence shows that specific rTMS protocols can modulate the activity of the various neurotransmitter circuits that are affected in AD.

## 5. Clinical Evidence for TMS in Alzheimer’s Disease

Over the last few years, clinical research on TMS in AD has evolved from promising initial exploratory pilot studies to randomized controlled trials employing network-based and personalized neuromodulation strategies [21,22,23]. These studies indicate that rTMS can improve or stabilize overall cognitive performance, modulate DMN brain networks, and result in neurophysiological and biomarker changes consistent with enhanced neuroplasticity. Although there is variability across centers in study design and stimulation parameters, there is an accumulating body of evidence that supports the clinical feasibility and therapeutic promise of TMS as an adjunctive intervention in AD.

### 5.1. Randomized Controlled Trials and Cognitive Outcomes

Randomized controlled trials (RCTs) indicate that rTMS can improve cognitive performance in patients with mild-to-moderate AD. The most widely used protocol is 2 weeks of everyday (5 days/week) treatment, followed by maintenance treatments (either 1 or 2 sessions/week) that could last up to 6 months.

Koch et al., 2025 [23] conducted a 52-week RCT (divided into a 2-week intensive course and a 50-week maintenance phase) in patients with mild-to-moderate dementia due to AD targeting the precuneus, demonstrating slowed cognitive decline measured by several neuropsychometric tests, such as CDR-SB, improved ADAS-Cog, MMSE, and improved functional outcomes (assessed by ADCS-ADL). Similar results were observed in the same study group, which followed the same experimental design for 24 weeks [64]. Functional MRI indicated increased hippocampal–precuneus connectivity, suggesting that rTMS improves cognition by modulating network function.

Jung et al., 2024 [22] studied 30 patients with either MCI due to AD or mild AD dementia by using a personalized hippocampal network-targeted rTMS protocol. They delivered 20 sessions over 8 weeks and stimulated the left parietal area. This approach led to statistically significant improvements in ADAS-Cog, in particular in the memory domain (*p* = 0.002), with fMRI showing improved hippocampal–precuneus functional connectivity. Effects appeared by week 8, and improvements persisted for at least 4 weeks after treatment ended. Other stimulation sites, such as the cerebellum, were also studied [65,66] with significantly improved cognition as measured by the CDR-SB score.

On the other hand, Moussavi et al. [67], in the largest sample size up to date of 156 participants, observed that TMS treatment applied to bilateral DLPFC failed to show more cognitive improvement than the sham group either immediately or 6 months after the intervention.

Earlier trials targeting the DLPFC and temporoparietal regions also reported improvements in memory, language, and executive function. Nguyen et al. [68] combined rTMS with cognitive training (NeuroAD protocol). In the NeuroAD protocol, rTMS was targeted over six brain areas that were thought to be impaired in AD, such as the right and left prefrontal cortex, right and left parietal cortex, and Broca’s and Wernicke’s areas. These target areas were identified by the Neuronix neuronavigation system based on the individual patient’s brain MRI. According to the results of the study, the cognitive gains (i.e., significantly improved ADAS-Cog score) persisted for up to approximately 8 months post-intervention. Similar results were observed in a recent study by Luo et al., 2026 [69] and a study of mild AD patients [70]. Moreover, a meta-analysis by Menardi et al. [71] compared the effects of cognitive training and found that only the combination of rTMS and cognitive training produced significant results for overall cognitive function, while rTMS alone showed no effectiveness. These results indicate that both cortical stimulation and network-targeted approaches can have clinically meaningful results.

### 5.2. Meta-Analyses and Systematic Reviews

Meta-analyses consolidate the effects of rTMS across studies. A 2024 meta-analysis of nine randomized controlled trials including 361 patients [72] showed an improvement in MMSE and ADAS-Cog scores immediately after treatment, which persisted one month later. High-frequency protocols (>10 Hz) targeting the DLPFC for ≥20 sessions resulted in better therapeutic results. Stimulation of the DLPFC, the most common stimulation target for improving cognitive function in patients with AD, was found to be most effective. Other meta-analyses including patients with AD and MCI [73,74,75] confirmed moderate-to-large effect sizes on global cognitive function, emphasizing the importance of frequency, total sessions and pulses, duration and stimulation site. These analyses underscore the potential of rTMS as a symptomatic intervention while highlighting the need for protocol standardization to optimize efficacy. A summary of randomized control trials on the role of TMS in AD treatment is presented in Table 1.

### 5.3. Network and Neurophysiological Effects

Network-level mechanisms are getting more emphasis in clinical studies. Personalized stimulation of the hippocampus and precuneus enhances functional connectivity within the DMN and the precuneus-hippocampal circuits. DLPFC stimulation was shown to be the most effective stimulation site for improving cognitive impairment in AD patients [64]. Moreover, EEG studies report decreased delta-band activity and normalized theta and gamma oscillations that correlate with improved cognitive function [78]. Targeting networks holds promise; better rTMS outcomes correlate with higher baseline intra-cortical connectivity, suggesting pre-treatment DMN regulation predicts treatment response [79].

### 5.4. Safety and Tolerability

rTMS is effective and well tolerated in older adults and people with AD. The most frequently reported adverse effects are transient headaches and local discomfort at the site of stimulation. Seizures are extremely rare, with a rate of roughly <1 per 30,000 sessions, when strictly adhering to established guidelines. When present, they resolve on their own [80]. Long-term follow-up, including the 52-week precuneus trial, found no sustained adverse effects, supporting the safety of repeated and maintenance sessions.

### 5.5. Biomarker-Guided Patient Selection for rTMS in Alzheimer’s Disease

Recent advances in blood-based biomarkers have enabled early and minimally invasive identification of individuals along the AD continuum. Among these, plasma phosphorylated Tau217 (p-Tau217) has demonstrated good accuracy in distinguishing amyloid-positive from amyloid-negative individuals across pre-clinical AD, mild cognitive impairment (MCI), and dementia stages [81,82]. This development shows promise for neuromodulation therapies such as rTMS.

The majority of rTMS trials to date have enrolled patients based on clinical diagnosis, without taking into consideration the underlying pathology. However, AD is now considered a biological continuum where DMN dysfunction precedes structural degeneration and clinical symptoms. Since rTMS primarily acts by modulating synaptic plasticity and network connectivity, its therapeutic potential is likely maximal before extensive neurodegeneration occurs, during stages characterized predominantly by synaptic and network dysfunction. Plasma p-Tau217, in combination with amyloid biomarkers, could be used as a practical tool to identify patients at this disease stage who are more likely to respond to neuromodulation.

Therefore, this biomarker-guided patient stratification would shift rTMS research towards precision neuromodulation and could be incorporated in clinical trial design, increasing the possibility of achieving maximal therapeutic effects.

## 6. Discussion

TMS offers non-invasive AD neuromodulation, potentially improving cognition and influencing disease networks and pathology. TMS helps restore synaptic plasticity, normalizes network connectivity, and reduces neuroinflammation; it may indirectly influence beta-amyloid and tau protein levels. Neurovascular and metabolic improvements further support neuronal resilience.

Current clinical evidence suggests that rTMS, particularly when delivered in personalized, network-targeted protocols, can improve memory and global cognition in patients with mild-to-moderate AD, with effects lasting in some protocols up to 3 months [67]. rTMS demonstrates a good safety profile in AD treatment, with multiple studies reporting few adverse events. Biomarker and neuroimaging studies suggest that TMS-induced improvements in connectivity and plasticity correlate with clinical cognitive improvements; this agrees with preclinical findings that TMS improves connectivity and plasticity in animal models. Current guidelines suggest that multisite rTMS, together with cognitive training, is probably effective (Level C of Evidence) in improving cognitive function in AD patients, especially at a mild/early stage of the disease [16].

A comparative study of the clinical trials summarized in Table 1 reveals that reported cognitive outcomes are influenced by specific rTMS parameters. Studies using high-frequency stimulation (10–20 Hz) of the DLPFC for ≥20 sessions most consistently report improvements in global cognition and memory measures (as assessed by global cognitive tests ADAS-Cog, MMSE and episodic memory tasks). In contrast, studies using low-frequency protocols, fewer sessions, or single-site stimulation more frequently report null or modest effects. A second parameter is the stimulation site. Multisite protocols targeting DMN areas such as DLPFC combined with precuneus or inferior parietal cortex tend to report broader cognitive benefits compared to single-site stimulation. These findings align with the concept of AD as a network disconnection syndrome and suggest that network-based targeting may be more effective than focal stimulation. Another factor is the use of cognitive training in combination with rTMS. Studies integrating stimulation with task-based cognitive engagement more often demonstrate positive outcomes, suggesting that rTMS may act by enhancing activity-dependent plasticity. While several studies report short-term cognitive improvements following rTMS, others failed to show positive results that might be due to variability in patient characteristics, disease stage, and study design. Potential explanations include small sample sizes, differences in outcome measures, different disease severity, and lack of biomarker-guided patient selection. Heterogeneity in stimulation protocols, target selection, session duration, and patient characteristics limits cross-study comparisons and definitive conclusions regarding TMS efficacy in AD. Larger, multicenter trials with extended follow-up and integration of multimodal biomarkers (e.g., resting state connectivity, TMS EEG, PET imaging, fluid biomarkers), genotype stratification (e.g., BDNF, APOE), and longer follow-up are required in order to assess the long-term effects and potential disease-modifying potential of TMS [83]. There is currently no evidence that rTMS modifies the underlying disease process or produces sustained benefits over the years.

Future research should focus on target precision neuromodulation using imaging and physiological markers in order to design individualized TMS protocols. Combining TMS with cognitive training, pharmacotherapy, or other methods of neuromodulation may further enhance therapeutic outcomes. Continued exploration of TMS mechanisms will elucidate how network, glial, and synaptic modulation contribute to disease modification, ultimately bridging preclinical insights with clinical application.

Compared to other recently published reviews [31,84], this narrative review provides a structured comparative analysis of stimulation parameters across studies, as well as differentiates between symptomatic network enhancement and potential disease-modifying effects. Moreover, a biomarker-guided stratification model is proposed to incorporate plasma biomarkers such as beta-amyloid and plasma p-Tau217.

In summary, TMS holds significant promise as a safe and potentially disease-modifying adjunctive therapy for AD. Its clinical role will depend on whether future research can demonstrate reproducible and long-lasting cognitive benefits using standardized protocols. Ongoing advancements in personalized stimulation protocols and mechanistic understanding are poised to optimize its clinical effectiveness and integrate TMS into new multi-modal therapeutic strategies that will target cognitive impairment and network dysfunction in AD.

## Figures and Tables

**Table 1:** Summary of randomized control trials on the role of TMS efficacy in AD treatment.

Study (Year)	Sample Size	Target Region	Frequency (Hz)	Sessions	Main Cognitive Outcomes	Notes/Biomarkers
Koch et al., 2025 [23]	60	Precuneus	10	52 weeks, weekly	Slowed decline in CDR-SB (1.36, *p* < 0.05), ADAS-Cog (5.9, *p* < 0.05); improvements in MMSE & ADCS-ADL	Functional MRI: increased precuneus-hippocampus connectivity
Jung et al., 2024 [22]	40	Hippocampal network (personalized)	20	8 weeks, 20 sessions	ADAS-Cog improvement (coefficient [SE], −5.2 [1.6]; *p* = 0.002); enhanced memory & attention	fMRI connectivity changes correlated with cognitive gains
Zhang et al., 2025 [65]	28	Cerebellum	3 pulses at 50 Hz, repeated at 5 Hz for 200 ms	4 weeks. 20 sessions	CDR-SB improvement (adjusted mean difference, −0.76)	
Koch et al., 2022 [64]	50	Precuneus	20	24 weeks, 32 sessions	Slowed decline in CDR SB (−0.25, *p* < 0.05), ADAS Cog (−0.67, *p* < 0.05); improvements in MMSE & ADCS ADL (*p* < 0.05)	
Yao et al., 2022 [66]	27	Cerebellum	5	4 weeks. 20 sessions	Increase in MMSE, MOCA, ADAS-Cog scores (*p* < 0.001)	
Moussavi et al., 2024 [67]	156	Bilateral DLPFC	20	4 weeks, 20 sessions	No ADAS-Cog improvement,	Similar results with Sham Coil
Sabbagh et al., 2021 [70]	109	6 brain areas	10	30 sessions	Average improvement on ADAS-Cog −2.11	
Nguyen et al., 2017 [68]	10	6 brain areas	10	25 sessions 5 weeks	MMSE & ADAS-Cog improvement	Combined with cognitive training (NeuroAD protocol)
Zhang et al., 2019 [76]	30	DLPFC + left temporal lobe	10	4 weeks, 20 sessions	ADAS-Cog and MMSE improvement (−3.52 ± 0.49),	Combined with cognitive training
Liu et al., 2024 [34]	75	DLPFC	20	30 sessions/6 weeks	Improvement in MMSE & ADAS-Cog	Correlation with plasticity scores
Mencarelli et al., 2024 [77]	16	Precuneus	20	10 daily, for 2 weeks, then 1/week for 22 weeks	Preservation of gray matter	

## Data Availability

Not applicable. No new data were created or analyzed in this study.

## Abbreviations

The following abbreviations are used in this manuscript:

TMS	Transcranial Magnetic Stimulation
AD	Alzheimer’s Disease
MCI	Mild Cognitive Impairment
DMN	Default mode Network
DLPFC	Dorsolateral Prefrontal Cortex
BDNF	Brain-Derived Neurotrophic Factor
ROS	Reactive Oxygen Species
